# Greenhouse Gases Life Cycle Assessment (GHGLCA) as a decision support tool for municipal solid waste management in Iran

**DOI:** 10.1186/2052-336X-12-71

**Published:** 2014-04-23

**Authors:** Rouhallah Mahmoudkhani, Behzad Valizadeh, Hamidreza Khastoo

**Affiliations:** 1 Islamic Azad University, Tehran medical Branch, Shariati St. Zargande, Tehran 14689-54311, Iran; 2 Environmental and Occupational Health centre, Ministry of Health and Medical, Education, Hafez St, Tehran, Iran

**Keywords:** Energy consumption, Greenhouse gas, Life cycle assessment, Municipal solid waste

## Abstract

**Background:**

One of the most problems in developing countries is the integrated waste management and the effects on Greenhouse Gases (GHG) emission, Life Cycle Assessment (LCA) is used in this paper as a decision supporting tool in planning Municipal Solid Waste (MSW) managements.

**Methods:**

In this paper the EPA’s Waste Reduction Model (WARM) that provide GHG emission factors for waste stream components that are based on life Cycle Inventory (LCI) framework were used and The MSW management methods comprised in seven scenarios.

**Results:**

The amount of GHG which was generated from Iran’s waste sector estimated about 17836079 Metric Tons of Carbon dioxide Equivalents (MT CO_2_e) in this study. The lowest amount of GHG was generated by LFG capture system with energy recovery (557635 MT CO_2_e), while Incineration of materials being sent to landfill (1756823 MT CO_2_e), Landfill Gas (LFG) capture system with flaring (2929150 MT CO_2_e) and Improved source reduction and recycling (4780278 MT CO_2_e) emitted fewer GHG than the other scenarios. Lowest levels of gross energy consumption occur in source reduction with recycling and composting (-89356240 Mega British Thermal Unit, M BTU), recycling and composting (-86772060 M BTU) as well as Improved source reduction with recycling and composting (-54794888 M BTU).

**Conclusions:**

It appears that recycling and composting each offer significant GHG emissions and energy consumption reductions (scenarios 4, 5 and 6). Upon of the GHG emission and energy consumption results concluded that improved source reduction and recycling scenario has been the Balanced and appropriate technology for handling the solid waste streams in municipalities.

## Background

Waste sector is a significant contributor to greenhouse gas (GHG) emissions accountable for approximately 5% of the global greenhouse gas [1]. This portion consists of methane (CH_4_) and carbon dioxide (CO_2_) which emitting from anaerobic decomposition of solid waste and leachate decomposition respectively [1]. However, for a more holistic approach, streamlining life cycle activities should also be accounted when quantifying a Waste Management (WM) strategy impact on GHG emissions. The most common WM strategy today is landfilling and is expected to increase due to developing countries’ approach in moving away from open dumping wastes to landfilling [2,3].

Life Cycle Assessment (LCA) has been defined by Society of Environmental Toxicology and Chemistry (SETAC) as an objective process to evaluate the environmental burdens associated with a product, process or activity, by identifying and quantifying used energy and materials and waste released to the environment, and to evaluate and implement opportunities to environmental effects improvements [4]. Life Cycle Assessment (LCA) is being used as a tool for comparative evaluation. It has been used extensively to evaluate solid waste management systems and comparing of different scenarios for integrated waste management systems [5-7]. Use of LCA in decision making is also well-established [7-9].

According to the International Standard Organization (ISO) 14000 series (14041e43), a typical LCA study consists of the following stages: goal and scope definition; life cycle inventory (LCI) analysis, with compilation of data about energy and material flows and on emissions to the environment, throughout the life cycle of the case study (ISO 14041); assessment of the potential impacts (Life Cycle Impact Assessment, LCIA) associated with the identified forms of resources used as well as environmental emissions (ISO 14042); interpretation of the results from the previous studies in relation to the objectives of this study (ISO 14043) [7-10].

Although regulation on Solid Wastes Managements as an act and its executive guidelines was codified by the Ministry of Interior with the cooperation of relevant sectors to improve the solid waste management situation in Iran, however, municipal solid waste management is still a considerable problem in Iran.

Information on Municipal Solid Wastes Managements (MSWM) in Iran since 2002 was collected by the Iran Municipalities and Rural Organization [11] as a responsible agency in urban and rural solid waste sector. These data is contained information on the current solid waste management practices in municipalities, solid waste composition, generation, quantities and qualities of household and commercial institution’s solid wastes, etc. According to the latest study conducted in 2008, solid waste is being collected as 16.59 million tons/year by municipalities with exclusive solid waste collection services in Iran while 83% of this volume is disposed to landfills and most of the rest in dump sites of the municipalities [11]. For small landfills, Methane can be collected and flared. However, for large landfills, collected methane gas can be used to generate electricity and/or heat. Although in many of the municipalities, solid wastes were not generated enough as much to have an economically feasible Landfill Gas-To-Energy (LFGTE), but if several adjoining municipalities sent their waste to a centralized landfill then such a project would be feasible. However, a centralized landfill would increase transportation requirements for collecting and transferring waste to landfill [7].

In particular, the broad perspective of LCA makes possible to take into account the significant environmental benefits that can be obtained through different waste management processes. For instance, waste incineration with energy recovery reduces the need for other energy sources. Materials generated from recycling processes replaces production of virgin materials and biological treatment may reduce the need for production of artificial fertilizers and transportation fuel [10]. Some models, which are able to perform LCA of waste management systems also exist, with the purpose of speed up the analysis and allow the analysts to know how the changes in the system affect the environmental impacts through scenario analysis [12]. The methodological framework used in this paper is the LCA as defined by ISO standards (ISO 14040, 14043), with several methods jointly applied in order to investigate system performances under different points of view, such as material and energy requirements, environmental impacts and ecological footprint. Such approach is applied because the LCA of a product or service should be the assessment of the product with regard to its impacts on the environment and on human health, and should aim to be an overall ecological assessment. Therefore, a range of specific and selected environmental impacts is assessed, but other aspects such as economic and social factors are not considered [10].

LCA has lots of offers in terms of selection and application of suitable MSW management techniques, technologies, and programs to achieve specific waste management objectives. The objective of this study is to use the LCA as a tool to compare different solid waste management system options and determine the most feasible system for Iran. To this purpose, seven different scenarios of municipal solid waste management systems (MSWMS) that include different municipal solid waste processing and/or disposal methods (MSWP-DMs) were developed and, then, compared to each other with respect to their environmental impacts by using the Integrated Waste Management (IWM) approach.

## Methods

### Choice of integrated waste management model

There are several models to determine the best options for reducing GHG emissions from waste in Iran. The model’s outputs are in the form of Excel spreadsheets, and include a summary of the input and outputs data, including the total life-cycle emissions of GHGs and criteria pollutants.

For MSW management, united state environmental protection agency (USEPA) has conducted a streamlined life cycle inventory (LCI) focusing on the GHG impacts of ten MSW components (e.g., paper, plastics, metals) in various ways and the EPA’s Waste Reduction Model (WARM) [13] provide GHG emission factors for waste stream components that are based on a LCI framework, WARM calculates GHG emissions for baseline and alternative waste management practices, including source reduction, recycling, combusting, composting and landfilling. Additionally the model calculates energy use for each of the options.

In order to model the GHG emissions from the solid waste sector using the IWM model, a set of inputs is required. These are including:

(1) Categorization of the waste stream (how much waste is generated, how much of each category of waste is sent for landfilling, recycling, etc.);

(2) Knowledge of the destination of the wastes, i.e. how much of each component of the waste stream is recycled, composted, incinerated, digested an aerobically, or landfilled;

(3) For recyclables, the distance from materials recovery facilities to markets;

(4) For landfilled, incinerated and digested wastes, the average distance travelled to the site of waste treatment and disposal;

(5) For a landfill, the extent of LFG capture and ultimate use of LFG (flaring, conversion to energy);

(6) For compostable materials, their distance to the composting site [14].

### Scope definition

Seven different scenarios of MSWM that include different municipal solid waste processing and/or disposal methods (MSW-PDMs) were developed and, then, compared with respect to their environmental impacts. Environmental impacts of MSW-PDMs were evaluated by considering their water emissions, air emissions, final solid waste produced and energy consumption. The MSWMS scenarios were developed base on the current MSWMS that widely applied in Iran, and the standard MSWMSs which were applying in the world. The assessment of these scenarios will provide to compare different possibilities for the waste management system of Iran, so that environmental sustainability could be achieved. The management system components or MSW-PDMs considered in the scenarios were: transportation of MSWs, source reduction, material recovery facility (MRF), incineration, and landfilling.

### Life cycle inventory and system boundaries

**Figure 1:**
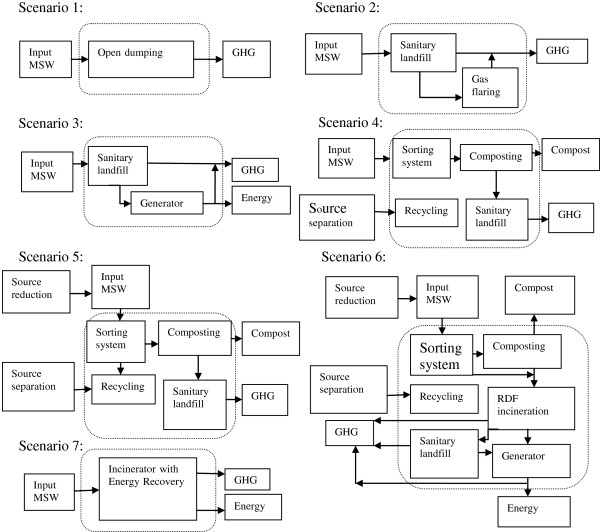
Schematic system flow diagrams for the different scenarios.

The data collection and preparation for Iran were mainly based on the projects prepared by Iran Municipalities and Rural Organization for Solid Waste Management System and recycling feasibility in Iran. These data include the population projections, the waste characteristics and composition, waste management applications, the comparison of the recommended transfer stations and landfill sites, the cost calculations for all the alternatives and operational recommendations for the landfill site. Application of averaged national data may not accurately reflect local conditions; however, in the absence of local data, national data is a good proxy. Composition and quantity of the solid waste produced in Iran is given in Table 1.Bearing in mind the constraints which are likely to exist in developing countries, a number of options were considered for the management of urban waste (Figure 1). The area which is outside of the dotted line shows resource and emission flows that may be generate or offset GHG emissions in the full system boundary. The description of the various scenarios clarifies what has been included in calculating the total GHG emissions for each scenario to enable equitable comparison. The inputs are collected non segregated wastes and therefore both of these options assuming use of a central facility. It was also assumed that the introduction of new facilities would leave the upstream scavenging operations largely unaffected.

**Table 1:** Iran municipal solid waste composition

**Waste component**	**Average (%)**	**Variation rate (%)**
Glass	1.91	1.05
Metals	2.34	1.38
Wood	1.27	0.9
Greens and food wastes	72.9	8.8
Paper	7.25	2.3
Plastics	8.4	3.1
Rubbers	1.09	1.01
Textiles	2.37	1.09
Oversize	3.6	2.7
Density (kg/m3)	253.73	52.5

As noted earlier, to assess the impact on the carbon balance, a life-cycle approach is needed and a systems boundary adopted to permit equitable comparison.

Where appropriate, activities common to all systems were ignored, e.g., although offsetting GHG emission benefits of materials reuse/recycling are highly positive, it was assumed that any minor changes (e.g., location) of materials recycling activities had minimal impact on the carbon balance and these activities were ignored. Further it was assumed that the precise location of facilities (and hence transport distances and modes) had minor impact on the carbon balance compared to the management practices adopted. Capital resource inputs were ignored in this study and consumable inputs were only considered where data were readily available and they were considered to be potentially significant in terms of the carbon balance [12].There are several scenarios that can be considered for modelling to determine the impact of different waste management strategies. For the purpose of determining the impact of different waste management strategies we considered seven scenarios for modelling as follows (Figure 1):

(1) The most general method of SWM in Iran is “open dumping in a mix of unmanaged sites”, all waste sent to landfill, no LFG capture occurs (dry waste recycling and composting occur at rates 5.3 and 12% respectively) [11], this is the baseline condition for waste management.

(2) LFG is captured and flared; recycling and composting occur at rates 5.3% and 12% respectively.

(3) LFG capture system upgrade (LFG capture rate increases to 75%, energy conversion facilities are installed, at 35% energy conversion efficiency, recycling and composting occur at rates 5.3% and 12% respectively.

(4) Source separation of materials currently being recycled, without improvements at any other point in the system (capturing 50% of the recyclable/compostable material that are currently not being captured);

(5) Source reduction of recyclable materials by 3.3% with source separation and recycling and composting of materials by 50%.

(6) Improved source separation and source reduction by 3.3%, recycling by 5.3% with organics diverted sent for composting by 59%, landfilling with energy recovery 32%, and using of Refuse Derived Fuel (RDF) production consists of sorting process in cement factory and RDF incinerators.

(7) Incineration of materials being sent to landfill, with energy recovery.The initial (base case) scenario is based on some simple assumptions about MSW management activities in the current year, Figure 1; case 1 displays the inputs for the current scenario (base case), the future scenarios assumes the state implements a set of MSW management activities designed to achieve a higher GHG emission and energy consumption reduction.

## Results and discussion

**Figure 2:**
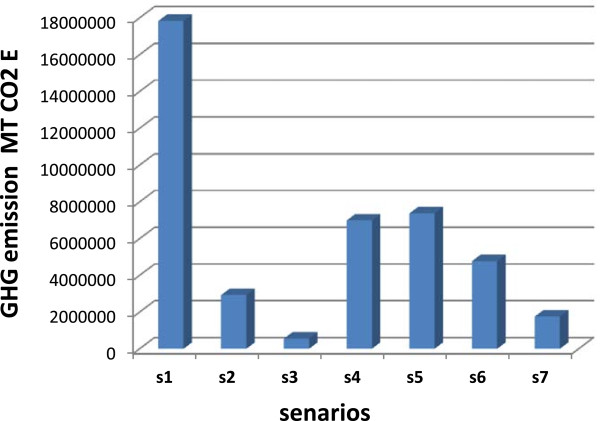
GHG emission for the different scenarios.

**Figure 3:**
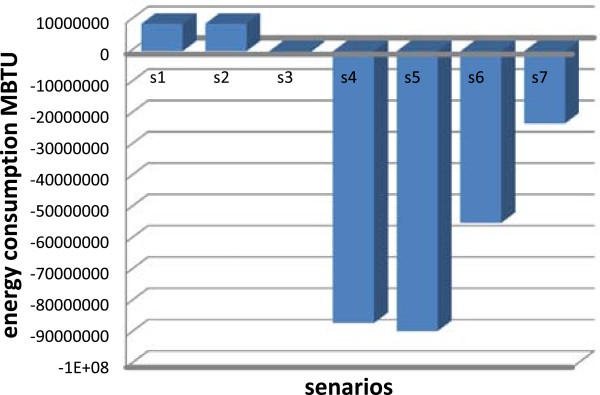
Energy consumption for the different scenarios.

The results displayed in this section are indicative of the GHG emissions and energy consumption statistics that may be possible through the seven scenarios analyzed. They should not be assumed to be exact, due to the assumptions necessary in the model and the actual conditions that are present in waste management in Iran. The emissions generated from the base case (the current state of waste management in Iran), for which the inputs are shown in Figure 1. The results of the modelling are displayed in the following figures. Figure 2 demonstrates the gross emissions from each scenario, by greenhouse gas, while Figure 3 displays the energy consumption of each scenario, However, for gross GHG emissions, the lowest amount was generated by scenario 3 (LFG capture system with energy recovery) (557635 MT CO_2_e), while scenario 7 (Incineration of materials being sent to landfill) (1756823 MT CO_2_e), scenario 2 (LFG capture system with flaring) (2929150 MT CO_2_e) and scenario 6 (Improved source reduction and recycling) (4780278 MT CO_2_e) also emitting fewer GHGs than the other cases considered. It should be noted that these figures only consider the emissions from the generation of waste by consumers, handling of waste by collectors, and disposal of waste. The model does not consider all emissions throughout the life-cycle of the materials. Surprisingly, the source reduction with recycling and composting scenario (S 5) (7373997 MT CO_2_e) does not demonstrate lower emissions than the recycling and composting scenario (S 4) (emissions are 6990088 MT CO_2_e).

It is also interesting to determine how much energy is being consumed in each of the scenarios, as energy consumption is linked to greenhouse gas emissions. Most emissions recognized by the model result from energy consumption, including CO_2_, CH_4_ and N_2_O emission. The exceptions to this are LFG emissions. Figure 3 demonstrates the energy consumed through the waste management cycle excluding energy consumed by material recycling and energy displaced by avoiding the use of virgin materials through recycling. The consideration of energy consumed through the reprocessing of recycled materials account for the much higher positive energy consumption totals in Figure 3. The energy consumed through reprocessing recycled materials in scenarios 4, 5 and 6 was between 5 and 10 times greater than the energy consumed in waste management for scenarios 1, 2 and 3 though this energy consumption is more than compensated for by avoided energy consumption through the production of materials from virgin feedstock. As expected, the lowest levels of gross energy consumption occur in scenario 5 (source reduction with recycling and composting) (-89356240 M BTU), scenario 4 (recycling and composting) (-86772060 M BTU) and scenario 6 (Improved source reduction with recycling and composting) (-54794888 M BTU), respectively. In scenario 7, the incineration of 16.59 million tons/year of waste generates 23021450 M BTU. With this, it can be estimated that the rate of energy production is 1.47 MJ/kg of solid waste. At 75% energy conversion efficiency, as assumed in the inputs into the model, the energy content of the waste is 2 MJ/kg. It would be expected that there would be an overall output of energy for the cases where energy is generated. As for net energy consumption, recycling has an evident impact on reducing energy consumption (Figure 3). With the exception of the incineration case, scenarios 4, 5 and 6 (greater diversion of manufactured goods) displaces the greatest amount of energy consumption. From an emissions and energy consumption standpoint, it is evident that the incineration case reduces emissions to a greater extent than any other case. However, the negative aspects of incineration of waste, especially air pollution and cost, as well as the attendant difficulty of sitting an incinerator in Iran, make this option unattractive. The costs of these options are beyond the scope of this project. Composting, improved LFG capture, improved diversion and source reduction also offer reduced energy consumption and emissions. Using what was shown above and the information contained in the IWM model. The difference in energy consumption between the recycling and composting scenario (S 4) and the source reduction and recycling and composting scenario (S 5) is about 2584180 M BTU. This amount between the improved source reduction and recycling (S 6) and source reduction and recycling scenario (S 5) is about 34561352 M BTU. The difference in GHG emission between the source reduction and recycling scenario (S 5) and improved source reduction and recycling scenario (S 6) is about 2593719 MT CO_2_e.

The modelling scenarios analyzed in this paper indicate that emissions are reduced most through diverting 47% more of the waste that is currently recycled. Though this may seem a challenge, there already has been a noticeable increase in diversion rates in Iran; composting increased from 12% to 59% (capturing 47% of what was being discarded). A law banning the mix collection of organic material would increase the amount of organic material being diverted, making composting more feasible emissions and energy consumption reduction strategy. The benefits of composting in the low per-tone cost for GHG reductions. Improved LFG capture reduced GHG emissions by 557635 MT CO_2_e, as well as exporting energy. It appears that recycling and composting each offer significant GHG emissions and energy consumption reductions.

Iran’s waste sector generated 17836079 MtCO_2_e GHG in 2002, of which main percentages of that was produced by the decomposition of organic wastes in landfills. The main options for Iran’s solid waste sector to contribute to reducing emissions are through source reduction, source separation, recycling, LFG capture, composting, and incineration. LFG capture, composting, and incineration will directly reduce emission from landfills, while source reduction, source separation and recycling will indirectly reduce emissions, perhaps to a greater measure, through displacing the processing of virgin materials. The IWM model was used to analyze the emissions from the waste sector. It indicated that further diversion of materials through source reduction and recycling has the greatest effect on reducing GHG emissions, mostly through reducing production emissions through feeding recycled material instead of virgin material to production processes. LFG capture with energy recovery follows with the next highest levels of GHG emissions reductions, followed by incineration. Energy consumption is reduced most through source reduction and recycling, followed by incineration with energy recovery and improved LFG capture.

## Conclusions

The IWM underestimates the GHG emissions and energy consumption reductions on improved source separation and source reduction by 3.3%, recycling by 5.3% with organics diverted sent for composting by 59%, landfilling with energy recovery and using of RDF production consists of sorting process in cement factory and RDF incinerators by 32.4% (scenario 6). It is possible that scenario 6 will reduce emissions and energy consumption significantly and further efforts to divert recyclable materials will achieve the greatest amounts of emissions reductions in Iran’s solid waste sector.

## Competing interests

The authors declare that they have no competing interests.

## Authors' contributions

RM has participated in all stage of the study, design of the study, data analysis and manuscript preparation. HK carried out statistical and technical analysis of data intellectual helping for analyzing of data. BV performed data collection and carried out manuscript preparation. All authors read and approved the final manuscript.
